# Editorial: AI for Robot Modeling, Path Planning, and Intelligent Control

**DOI:** 10.3389/frobt.2020.00019

**Published:** 2020-02-20

**Authors:** Yongping Pan, Basil M. Al-Hadithi, Chenguang Yang

**Affiliations:** ^1^School of Data and Computer Science, Sun Yat-Sen University, Guangzhou, China; ^2^Intelligent Control Group, Technical University of Madrid, Centre for Automation and Robotics, UPM - CSIC, Madrid, Spain; ^3^Department of Electrical, Electronics, Control Engineering and Applied Physics, Technical University of Madrid, Madrid, Spain; ^4^Bristol Robotics Laboratory, University of the West of England, Bristol, United Kingdom

**Keywords:** Gaussian process, reinforcement learning, path planning, human-robot interaction, neural networks, collaborative robot

Artificial intelligence (AI) is intelligence demonstrated by machines, in contrast to the natural intelligence demonstrated by humans. Examples of AI research include reasoning, knowledge representation, planning, learning, natural language processing, perception, and the ability to move and manipulate objects, which is usually regarded as intelligent control. In recent years, the applications of AI to robotics have experimented with exponential growth. AI plays a crucial role in the path planning of robots, allowing fast responses to changes in complex environments. It also plays a leading role in modeling and intelligent control of robots by allowing a more complex feedback analysis, self-tuning applications, and on-the-fly adaptation to environmental changes.

Changing industrial environments like flexible manufacturing facilities and automated warehouses where robots are intended to work side by side with humans are benefiting directly from advancements in complex path planning and autonomous decision making based on AI-powered algorithms. On the consumer side, applications like cleaning robots and delivery robots are also becoming part of our daily lives. The implementation of AI-powered path planning and control algorithms drastically improves the efficiency and practicality of these robots, as the environments in which these robots must operate is highly dynamic and needs constant adaptation.

This Research Topic is organized under the section “Robotic Control Systems” within Frontiers in Robotics and AI. The first article by Tan et al. is focused on designing mechanisms and algorithms for robotics, which serves as a platform for path planning and control. Current robot designs have been taking inspiration from games and entertainment artifacts (GEAs). However, there is a lack of systematic and general processes for implementing a GEA-inspired design in robotics. In this article, a systematic robot design paradigm is proposed based on the inspiration of GEAs. Both problem-driven and solution-driven processes can be followed to make use of analogies of GEAs so that robotic solutions can be obtained for real-world problems. The application of the design paradigm is demonstrated by using a reconfigurable floor cleaning robot and its path planning algorithm.

Due to the capacity of reasoning, AI plays a crucial role in achieving safe human-robot interaction (HRI) for collaborative robots. The article by Du et al. combines different AI technologies to achieve active collision avoidance for safe HRI. A Microsoft's motion sensing input device named Kinect is employed to detect anyone who enters the workspace of the robot so that the skeleton data of the human can be calculated in real-time. An expert system with collision avoidance knowledge is employed to analyze the behavior of the human for active collision avoidance. An artificial potential field method is adopted to plan a new path for the robot such that it can bypass the human in real-time. Experiments show that by applying these AI-powered algorithms, the proposed system can safeguard the human by detecting the human and analyzing the motion of the human.

An important issue for collaborative robots is to learn the compositionality of human activities, i.e., to recognize both activities and their comprising actions. Even a small set of actions and objects can create a large combination of possible activities. Most existing approaches in this topic address action and activity recognitions separately. The article by Mici et al. suggests learning human activities concurrently on two levels of semantic and temporal complexity: Transitive actions such as reaching and opening a cereal box, and high-level activities such as having breakfast. The learning model consists of a hierarchy of growing-when-required (GWR) networks which can process and learn inherent spatiotemporal dependencies of multiple visual cues abstracted from human body skeletal representation and interaction with objects. GWR means that new network nodes are added only when the number of iterations is an integer multiple of some pre-defined constant. The proposed architecture semantically segments input RGB-D sequences of high-level activities into their component actions without supervision. Experiments show that the proposed approach possesses a superior ability regarding the classification of high-level activities.

AI is also useful for collaborative robots to assist humans in co-manipulation and teleoperation tasks under demonstrated trajectories. Most existing approaches in this topic are not applicable when the solutions of demonstrations are suboptimal or when the generalization capabilities of the learned models cannot cope with changing environments. The article by Ewerton et al. presents a reinforcement learning-based approach to solve the problem above. The proposed approach makes use of the concept of relevance functions and is initialized by a probability distribution of demonstrated trajectories. Gaussian Process regression is applied to cope with the changes in dynamic environments. Experiments demonstrate that the proposed algorithm embedded in a 7-degree of Freedom (DoF) robot arm performs well under a dynamic environment.

The last article by Galati and Giulio serves more as an outreach of this Research Topic, where a physical model-based approach for terrain characterization is presented for a tracked skid-steer vehicle. A set of physics-based parameters, including the equivalent track, the power spectral density for the vertical accelerations, drive motor electrical currents, and motor currents, is employed to characterize the terrain properties. The proposed algorithm predicts the type of terrain that the robot traverses based on the parameter set. Experiments under various surfaces verify the effectiveness of the proposed approach for autonomous robots. The results of this article also indicate that the intelligent integration of model-based and AI-based techniques will be promising in robotic applications.

## Author Contributions

All authors listed have made a substantial, direct and intellectual contribution to the work, and approved it for publication.

### Conflict of Interest

The authors declare that the research was conducted in the absence of any commercial or financial relationships that could be construed as a potential conflict of interest.

